# Posterior Reversible Encephalopathy Syndrome due to Hypomagnesemia: A Case Report and Literature Review

**DOI:** 10.1155/2018/1980638

**Published:** 2018-11-29

**Authors:** Mohamad Almoussa, Angelika Goertzen, Stephan Brauckmann, Barbara Fauser, Christoph W. Zimmermann

**Affiliations:** Department of Neurology, St. Josef Hospital, The Academic Hospital of Duisburg-Essen University, Mülheimer Strasse 83, 46045 Oberhausen, Germany

## Abstract

**Background:**

Hypomagnesemia can cause various unspecific neurological complications, which can lead to diagnostic confusion. One of these complications is the posterior reversible encephalopathy syndrome (PRES), which is extremely uncommon and has been reported only twice in the English-language literature.

**Case presentation:**

We report the case of a 60-year-old man who presented with PRES involving only the cerebellar hemispheres and associated with hypomagnesemia. After excluding all the other possible etiologies of PRES, we started magnesium replacement therapy, which led to a remarkable but fluctuating clinical and chemical improvement. A full recovery with no need for further supplementation was achieved only after discontinuation of a proton pump inhibitor.

**Conclusions:**

This case highlights the role of magnesium in the pathophysiology of PRES; thereby, underlying hypomagnesemia should be considered in every PRES case with unclear etiology.

## 1. Background

Magnesium is the second most abundant intracellular cation after potassium and the fourth most abundant extracellular cation overall. Ninety-nine percent of the magnesium is stored intracellularly, principally in the bone and to a lesser extent in the muscles. The plasma magnesium, which comprises only 1% of total magnesium, can be ionized or bound to anions or protein. Magnesium plays a crucial role in numerous physiological functions; therefore, its hemostasis in the body is strictly regulated by uptake in the small intestine and excretion in the kidney [1]. Hypomagnesemia is usually defined as having a magnesium level below 0.66 mmol/L (1.6 mg/dl) [2]. To avoid hypomagnesemia, the German Society for Nutrition recommends a sufficient daily intake. Common etiologies of hypomagnesemia include chronic inadequate intake, alcoholism, vomiting, and diarrhea. Other conditions associated with increased gastrointestinal magnesium loss include malabsorption, steatorrhea, short gut syndrome, pancreatitis, and genetic disorders affecting magnesium absorption. Similarly, hypomagnesemia may be the consequence of enhanced magnesium renal wasting caused by some medications (diuretics, EGFR inhibitors, calcineurin inhibitors, cisplatin, carboplatin, aminoglycoside antibiotics, pentamidine, rapamycin, and amphotericin B) and genetic disorders such as Bartter and Gitelman syndromes [1]. Proton pump inhibitors cause hypomagnesemia, probably by affecting its intestinal absorption [1]. Patients with mild magnesium deficiency may suffer nonspecific symptoms such as depression, tiredness, muscle spasms, and muscle weakness [1]. Critically low magnesium concentrations can cause serious complications such as cardiac arrhythmias, seizures, neuromuscular irritability, muscular weakness, and respiratory depression [3]. Hypomagnesemia may also lead to a wide spectrum of neurological disorders, such as primary downbeat spontaneous nystagmus with ataxia [4–6], cerebellar syndrome [7–9], myopathy [10], and posterior reversible encephalopathy syndrome (PRES) [11, 12]. Other symptoms such as depression, agitation, cognitive confusion, and coma have been also reported [13].

Here, we report a case of PRES in the setting of hypomagnesemia and provide a literature review on cerebellar symptoms attributed to it.

## 2. Case Presentation

A 60-year-old man was admitted to the internal department of our hospital due to thoracic discomfort, vertigo, nausea, and ataxia. After excluding acute coronary artery disease, he was referred to us because of the progression of the neurological symptoms during his one-week stationary therapy in the internal department.

On clinical examination, he demonstrated a remarkable limb and truck ataxia, a rest, postural, and intention tremor, a severe dysarthria, nystagmus, and a mild cognitive impairment. The patient could not walk or eat unassisted. His current oral medication consisted of acetylsalicylic acid, pantoprazole 40 mg/d, atorvastatin, spironolactone, opipramol, Ferro Sanol, and ramipril.

**Figure 1:**
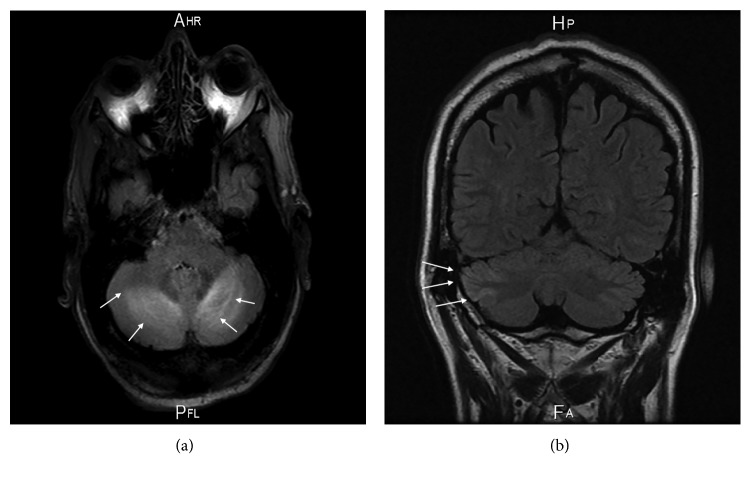
Cranial MRI displays high signal intensity in both cerebellar hemispheres six months prior to admission (a); and, currently, a residual hyperintensity in the right cerebellar hemisphere on T2-weighted and FLAIR images (b).

His medical history included hypertension, coronary artery disease, an episode of gastrointestinal bleeding by angiodysplasia in 2015, and pulmonary embolism in 2013. In addition, he had a medical history of persistent diarrhea over the last two years without any organic etiology, a vitamin D deficiency in spite of substitution, and recurrent hypokalemia. The patient was a habitual drinker consuming two glasses of wine daily. Six months ago, he was hospitalized in another neurological department because of a one-week persistent dysarthria. An obtained cranial magnetic resonance imaging (MRI) at that time revealed a symmetric hyperintensity in both cerebellar hemispheres (Figure 1(a)). To exclude a cerebellar paraneoplastic syndrome and viral or autoimmune encephalitis, a lumbar puncture was performed. The analysis result of the cerebrospinal fluid was normal. Antibodies against NMDA-receptors, AMPA1-receptors, AMPA2-receptors, and autoantibodies against Ma2 and M2, as well as herpes simplex antibodies (HSV1- and HSV2-DNA), were not detected in the cerebrospinal fluid. A computed tomographic scan of the thorax and abdomen was unremarkable. An empirical therapy with Rocephin and aciclovir was started, but after excluding herpes simplex in the cerebrospinal fluid, the antiviral therapy was discontinued. The blood pressure was slightly high during monitoring; therefore, an antihypertensive therapy was initiated. A further coloscopy and gastroscopy revealed only a *Helicobacter pylori*-negative gastritis. The dysarthria improved, and the patient was discharged with the diagnosis of a possible PRES according to the cranial MRI finding.

The laboratory investigations disclosed a severely low magnesium level (0.4 mg/dl; range: 1.7–2.55 mg/dl), a hypocalcemic level (1.7 mmol/l; range: 2.1–2.5 mmol/l), a normal potassium level (3.6 mmol/l; range: 3.5–5.1 mmol/l), a low hemoglobin count (12.3 g/l; range: 14–17.5 g/l), a low erythrocyte count (3.69 × 10^6^/*µ*l; range: 4.5–5.9 × 10^6^/*µ*l), a low 25-OH vitamin D level (7 ng/ml; range: 31–100 ng/ml) despite the replacement therapy, and a normal parathormone (PTH) level (22.3 pg/ml; range: 14.5–87.1 pg/ml). Sodium and phosphate levels were within the normal range. The creatine kinase level was high (450 U/l; range <174 U/I). The other laboratory tests including serum electrophoresis were within the normal range. During the stationary therapy, he developed a mild hypokalemia; an oral supplementation was started.

The cranial MRI displayed a weak residual hyperintensity in the right cerebellar hemisphere, probably as a residual indicator of the cerebellar bihemispheric hyperintensities described in the previous external MRI (Figure 1(b)). The electroencephalography results were normal. To exclude a paraneoplastic syndrome, we performed a lumbar puncture, which revealed an unremarkable finding. Another possible cause for PRES such as high hypertension was missing. Thus, we suspected the cerebellar syndrome due to hypomagnesemia and started an intravenous magnesium supplementation and an oral calcium intake.

The patient received an intravenous supplementation of 1 g magnesiumsulfat-heptahydrat (equivalent to 4.05 mmol/mg) every two days, in addition to oral supplementation of calcium and potassium.

**Figure 2:**
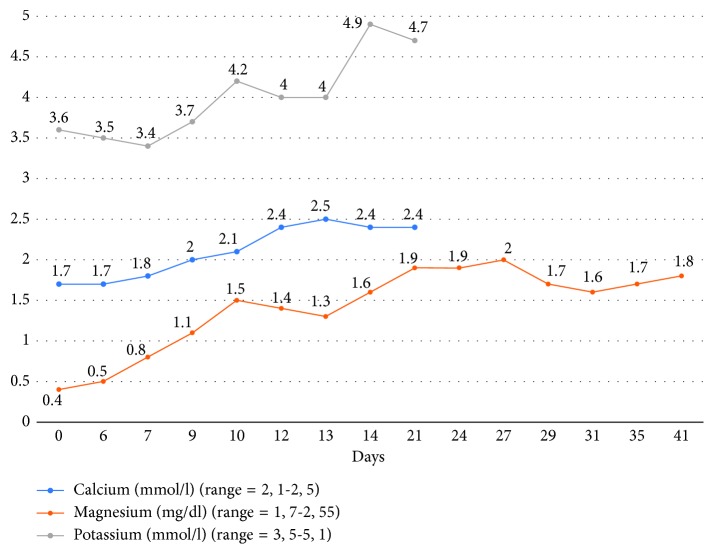
Serum magnesium, potassium, and calcium during the stationary therapy. At day 14, with a magnesium level of 1.6 mg/dl, the patient clinically remarkably improved and was discharged.

The magnesium level returned to the normal range after two weeks of supplementation, as did the calcium level within four days. The patient exhibited a clear clinical improvement of the ataxia; he could walk and eat unassisted (Figure 2). After 14 days of hospitalization, the patient was discharged. The patient received poststationary magnesium intravenous supplementation three times per week for two months. Notably, excreted magnesium in the 24-hour urine specimen was normal excluding the renal waste of magnesium. However, fluctuations in magnesium levels and the clinical symptoms were still observed under the poststationary intravenous supplementation until the proton pump inhibitor (PPI) was discontinued and a therapy with ranitidine was started. Subsequently, the replacement therapy was discontinued. The patient has remained symptom-free for over five months.

## 3. Discussion

Posterior reversible encephalopathy syndrome is a neurological syndrome characterized by clinical and radiological features. It encompasses heterogeneous etiologies sharing similar findings on imaging studies. Although the pathophysiology underlying PRES remains unclear, it is believed that it is related to disordered cerebral autoregulation and endothelial dysfunction leading to a vasogenic edema in the posterior cerebral regions [14]. PRES due to magnesium depletion is rare and has been described, in just two case reports [8, 9]. In the present case, MRI studies revealed a PRES depiction involving only the cerebellar hemispheres. Isolated cerebellar involvement in the setting of PRES is extremely rare [15]. Various conditions associated with PRES have been identified; these include blood pressure fluctuations, renal failure, immunosuppressive therapy, autoimmune disorders, and eclampsia [14]. In our case, a high blood pressure level was documented on admission to the internal department, which was rapidly decreased without any complications. In addition, during the previous external hospitalization, only a slightly high blood pressure level was documented although the radiological finding at that time suggested PRES. Therefore, and after ruling out another possible etiology for PRES, we suspected hypomagnesemia. This was confirmed in the chemical analysis that disclosed a particularly low magnesium level of 0.4 mg/dl (range: 1.7–2.55 mg/dl). This severe hypomagnesemia may have resulted from chronic diarrhea, the intake of PPI, the habitual alcohol consumption, or a combination of these factors. Our patient had been taking PPI for several years and suffered chronic diarrhea for the last two years. In addition, he used to drink two glasses of wine daily for many years. Notably, the magnesium level in the 24-hour urine collection was normal excluding the renal wasting. Only ending the use of the PPI led to a stable, normal magnesium level after discontinuing the supplementation.

Blood tests disclosed a severe hypomagnesemia, accompanied by hypocalcemia, mild hypokalemia, and a chronic vitamin D deficiency but a normal PTH level. Magnesium depletion is known to induce renal potassium secretion and cause hypocalcemia by impairing PTH production or inducing resistance against it on target organs [1]. In the present case, PTH was within the normal range despite the severe hypocalcemia indicating a secondary hypoparathyroidism due to the hypomagnesemia. But, in two reported cases, hypomagnesemia and hypocalcemia were associated with an elevated PTH [8, 11].

The parenteral replacement therapy of magnesium led to rapid clinical improvement. Even before reaching the normal range, the patient could resume his daily activities without assistance. After replacement of PPI with ranitidine, we ended the poststationary magnesium intravenous supplementation.

There are ten case reports in the literature describing cerebellar syndrome due to hypomagnesemia (11 patients, six men and five women; Table 1). In four cases, hypomagnesemia developed due to short bowel syndrome after surgery. Notably, and similar to our case, hypomagnesemia was overlooked in all the reported cases and diagnosed only during the second or third hospitalization after ruling out other possible causes. For example, a lumbar puncture and an investigation for malignancies were performed in 4 of the 11 patients due to the suspicion of a paraneoplastic syndrome. An antiviral therapy with aciclovir was initiated in two patients. Regardless of the supplementation regimes applied, full recovery or clinical improvement was observed in 8 of 11 cases; reoccurrence of the symptoms was reported in three cases. Altogether, hypomagnesemia represents a curable cause of cerebellar and PRES syndrome.

## 4. Conclusions

Hypomagnesemia can cause serious neurological symptoms, including cerebellar syndrome and PRES. Investigation of underlying hypomagnesemia should be considered in these disorders, especially in the presence of other laboratory disorders and long-term PPI therapy. Intravenous supplementation and replacement of the PPI could resolve the symptoms. This can save time and avoid costly unnecessary investigations.

## Figures and Tables

**Table 1:** Cerebellar syndrome due to hypomagnesemia in the literature.

Publication	Age (years)	Sex	Neurological symptoms on admission	Imaging	Mg	Na	Ca	K	PTH	Etiology	Follow-up
[4]	78	M	PDBN	CCT: n MRI: cerebrovascular chronic ischemia and slight cerebral and cerebellar atrophy	Not detectable	n	↓	n	N/A	Probably attacks of diarrhea caused by diverticulitis	PDBN disappeared after two weeks. A temporary reoccurrence was observed on day 17

[12]	61	F	Ataxia, paresthesia, cognitive impairment	Brain and cord MRI: n	1.5 mEq/l	N/A	N/A	N/A	N/A	TRPM6 mutation	Reoccurrence after 2 months

[8]	72	M	Severe dysarthria, ataxia, dysphagia, nystagmus	MRI: hyperintensities within both cerebellar hemispheres similar to PRES	0.15 mmol/l	N/A	↓	N/A	↑	Short bowel syndrome after surgical treatment of adenocarcinoma and diarrhea	MRI and clinic were unremarkable after 2 months

[11]	68	F	Seizure, PDBN	MRI: a lesion within the cerebellar nodulus	7 mg/l (range: 18–24 mg/l)	n	↓	↓	↑	Undetermined	Reoccurrence after 2 months

[7]	59	M	Ataxia, vertical nystagmus, seizures, PDBN	MRI: hyperintensity and swelling of the cerebellar nodulus	<0.08 mmol/l (normal range: 0.75–1.0 mmol/l)	↓	↓	↓	N/A	Short bowel syndrome after ileostomy due to ulcerative colitis	N/A

[16]	66	F	Dysphagia, diplopia, vertical nystagmus, weakness, cognitive impairment	N/A	0.21 mEq/l (range: 1.4–2.0 mEq/l)	n	n	n	N/A	Short bowel syndrome after colectomy due to metastases of cervix carcinoma	Symptoms improved, dysphagia resolved after 2 months

[17]	67	F	PDBN, ataxia	N/A	1.1–1.4 mmol/l (range: 1.5–2.5 mmol/l)	N/A	↓	N/A	N/A	Side effect of lithium carbonate	Symptoms resolved in 4 months

[5]	21	M	PDBN, ataxia, dysphagia, tachycardia, seizures	CCT: n	<1 mg/dl	N/A	N/A	↓	N/A	Parenteral nutrition, short bowel syndrome after ileocolectomy for Crohn's disease	Complete recovery after 6 weeks

[5]	44	F	Seizures, PDBS	CCT: n	0.9 mg/ml (range: 1.5–3.5 mg/dl)	N/A	↓	↓	N/A	Parenteral nutrition, resection of terminal ileum and cecum because of metastatic fallopian adenocarcinoma	Persistence of downbeat nystagmus; death because of cancer complications after 3 months

[9]	57	M	Seizure, dysarthria, ataxia	MRI: hyperintense lesions in both cerebellar hemispheres and the vermis resembling PRES	0.19 mmol/l	N/A	N	↓	N/A	Alcohol abuse	Significant improvement after 6 months

[18]	65	M	Ataxia, cognitive impairment, seizure	MRI: hyperintensities within the cerebellar vermis	0.08 mmol/l (range: 0.7–0.9 mmol/l)	N/A	↓	↓	↓↓↓	Pantoprazole	Mild memory deficit is still observed after 6 months

M: male; F: female; PDBN: paroxysmal downbeat nystagmus; n: normal; N/A: not available; Mg: magnesium; Ca: calcium; K: potassium; PTH: parathormone hormone; MRI: magnetic resonance imaging; CCT: cranial computed tomography. mEq/l: milliequivalents per liter; mmol/l: millimoles per liter; mg/dl: milligrams per deciliter; mg/ml: milligrams per milliliter. ↓: low; ↓↓↓: very low; ↑: high.

## Abbreviations

PRES: Posterior reversible encephalopathy syndrome
PPI: Proton pump inhibitor
EGFR: Epidermal growth factor receptor
MRI: Magnetic resonance imaging
NMDA-receptors: *N*-methyl-D-aspartate receptor
AMPA-receptors: a-amino-3-hydroxy-5-methyl-4-isoxazolepropionic acid-receptors
Mg: Magnesium
PTH: Parathormone.
